# Removal of Ochratoxin A from Grape Juice by Clarification: A Response Surface Methodology Study

**DOI:** 10.3390/foods11101432

**Published:** 2022-05-16

**Authors:** Majid Behfar, Ali Heshmati, Freshteh Mehri, Amin Mousavi Khaneghah

**Affiliations:** 1 Nutrition Health Research Center, Hamadan University of Medical Sciences, Hamadan 6517659947, Iran; majidbehfa0@gmail.com (M.B.); freshteh_mehri@yahoo.com (F.M.); 2 Department of Food Science and Nutrition, Faculty of Food Engineering, University of Campinas (UNICAMP), Campinas 13083-862, Brazil

**Keywords:** ochratoxin A, grape juice, clarification, antioxidant compounds, mycotoxin

## Abstract

This study achieved maximum removal of ochratoxin A (OTA) during the grape juice clarification process with minimal reduction in antioxidant compounds (phenolic acid, flavonoids, and antioxidant capacity by FRAP) by the RSM method. Independent variables included three types of clarifiers—gelatin, bentonite, and diatomite (diatomaceous earth)—at a concentration level of 0.25–0.75% and clarification time of 1–3 h. OTA was measured by high-performance liquid chromatography with fluorescence detection. Clarifying agent concentration and clarification time affected the reduction amount of OTA and antioxidant compounds in grape juice. There was a direct linear correlation between the reduction amounts of OTA and antioxidant compounds and capacity with the concentration of bentonite, gelatin, and diatomite, and the clarification time. The reduction amount of OTA and antioxidant capacity followed the linear mode. However, the decreased phenolic acid and flavonoid values followed the quadratic model. The study results showed that if the concentrations of bentonite, gelatin, and diatomite and clarification time were 0.45, 0.62, 0.25%, and 1 h, respectively, the maximum amount of OTA reduction (41.67%) occurred. Furthermore, the phenolic acid, flavonoid, and antioxidant activity decrease amounts were at their lowest levels, i.e., 23.86, 7.20, and 17.27%, respectively.

## 1. Introduction

Fruits and vegetables are the main components of the human diet, mainly cultivated in different regions [1]. Today, the tendency to consume fruit-derived products such as grape juice is growing because they are a good source of sugars (fructose, glucose), organic acids (tartaric acid, malic acid, citric acid), minerals (Cu, Fe, Mn), phenolics (catechin, malvidin 3,5-diglucoside, Cyanidin-3,5-diglucoside), and vitamins [2,3,4,5,6]. In addition, they contain various antioxidants, which play a vital role in human health and disease risk decreases, such as cancer and cardiovascular diseases [7,8]. In 2017, the global consumption of fruit juices was estimated at 35 billion liters [9].

The high sugar levels in grapes cause this product to be spoiled by various fungi, such as mycotoxin-producing molds [5,10]. Therefore, despite the critical role of grapes in humans’ diet and health, one of the greatest concerns is the contamination of these foodstuffs with different types of mycotoxins. The different types of mycotoxin include ochratoxin A (OTA), patulin, aflatoxins, citrinin alternariol, and tenuazonic acid, and fumonisin B2 might be created during fungi growth on grapes [11]. However, OTA is the most critical mycotoxin, and its occurrence has been reported in grapes and products obtained from grapes, such as grape juice, raisins, pekmez, and wine [12,13,14,15].

OTA is a secondary metabolite produced by specific fungi species, including Aspergillus and Penicillium [16]. OTA has also been categorized as a group 2B carcinogen for humans by the International Agency for Research on Cancer [17]. Various toxic effects of OTA on human health have been reported, such as teratogenic, carcinogenic, mutagenic, neurotoxic, hepatotoxic, and immunotoxic effects [14,18]. Furthermore, OTA causes various human nephropathies, including Balkan Endemic Nephropathy (BEN) and chronic interstitial nephropathy (CIN) in humans, and increases lipid peroxidation, inhibiting macromolecular synthesis and the inhibition of mitochondrial respiration [19,20].

The presence of OTA in grape-derived products is reported in many countries, where the content is related to cultivation, transportation, and storage [13,14,17,21]. Due to the high contamination of grape OTA, the maximum level for this mycotoxin in products such as wine, wine-based drinks, and grape juice is considered 2.0 µg/kg [22]. In order to remove OTA from contaminated foodstuffs, different strategies, such as physical, chemical, and biological methods, have been suggested [23]. The physical methods are uneconomical [24]. The residue of synthetic chemicals and fungicides applied for OTA control has harmful impacts on human health [25]. In biological methods, although metabolized OTA forms created by S. cerevisiae are less toxic, they may be converted to OTA in the digestive system [26].

The application of adsorbents is the most common practice for OTA reduction [27]. The reduction of OTA by bentonite, gelatin, and diatomite was reported in previous studies [12,28,29]. In addition to OTA removal, these clarifiers might decrease antioxidant compounds [30,31,32,33]. As antioxidant compound removal could decrease the nutritional value of grape juice, it is necessary to optimize the applied clarifier level to decrease the highest level of OTA. In contrast, antioxidant compound amounts did not change or had a low loss. This study aimed to optimize bentonite, gelatin, and diatomite levels and clarify the time for obtaining the highest removal of OTA and lowest reduction in antioxidant components and capacity.

## 2. Materials and Methods

### 2.1. Materials

A grape sample (Askari cultivar) was collected from a garden in Hamadan (Iran). OTA, 2,4,6-Tris(2-pyridyl)-s-triazine (TPTZ), Folin–Ciocalteu reagent, gallic acid, and catechin were supplied by Sigma (St Louis, MO, USA). Sodium carbonate, phosphate-buffered saline (PBS), sodium nitrate, methanol, sodium acetate, aluminum chloride, ferric chloride, sodium hydroxide, hydrochloric acid, acetonitrile, ferrous sulphate, and other chemicals were supplied by Merck (Darmstadt, Germany). Immunoaffinity columns (IAC) were bought from Libios (Pontcharra-Sur-Turdine, France). Bentonite was bought from Mojallali Inc. (Tehran, Iran). Gelatin by Diaco (Tehran, Iran) and diatomite earth were supplied by Neutron (Tehran, Iran). A Millipore Milli-Q purification system (Millipore, Milford, CT, USA) was used to prepare ultra-pure water.

### 2.2. Grape Juice Preparation

First, the OTA concentration in collected grape samples was measured according to the method mentioned below. The OTA content of grape specimens was lower than the limit of detection (LOD). Then, grape samples were washed. For washing, grape samples (5 kg) were immersed in 10 L of tap water for 10 min. Furthermore, they were placed on a steel strainer to dry. Then, they were crushed and pressed by juicing mashing (Pars Khazar, Tehran, Iran) to obtain grape juice.

For grape skin and seed removal, grape juice was filtered through Whatman No. 2 filter paper. For this study, OTA was spiked into filtered grape juice at a concentration of 5 µg/L. The initial concentration of OTA was considered fixed based on the instrument limitation.

### 2.3. Addition of Clarifier to Grape Juice

As shown in Table 1, three types of clarifiers, i.e., bentonite, gelatin, and diatomite earth, were added to grape juice at different levels (0.25, 0.5, and 0.75% *w*/*v*) and agitated for 10 min by a magnetic stirrer (MTOPS, HS15-03P model, Korea). Samples were placed at ambient temperature for different times (1, 2, and 3 h). Couples were passed through Whatman filter paper (No. 2).

### 2.4. OTA Clean-Up and Measurement

Before analysis, unclarified grape juice and clarified grape juice samples were stirred entirely. The OTA extraction and analysis method was similar to our previous study, with slight modifications [34]. First, methanol (25 mL), deionized water (160 mL), and NaCl (2.5 g) were added to 40 mL juice samples and they were stirred for 10 min by a magnetic stirrer. Then, they were filtered through Whatman No. 1 filter paper. Twenty-five mL of filtrated sample was mixed with PBS (125 mL). Samples were centrifuged at 1252 g, for 10 min (Hettich, Tuttlingen, Germany). Fifty mL of the centrifuged specimen was passed through IAC. OTA was eluted with methanol (1 mL). The volume of collected fluid was increased to 2 mL with methanol. In the final step, 50 µL was injected into the HPLC instrument.

### 2.5. Apparatus of OTA Analysis

OTA’s determination and quantity measurement was performed by an HPLC system (Milford, MA, USA) equipped with a binary pump and fluorescence detector (model 2475, Milford, MA, USA). OTA separation was carried out on a reversed phase C18 column (ODC) (250 mm × 4.6 mm, i.d., 5 µm) at 25 °C. The mobile phase utilized for OTA analysis was composed of a water, acetonitrile, and methanol mixture (5:3:2, *v*/*v*/*v*) and delivered to HPLC at a 1 mL/min rate under isocratic elution conditions. The measurement of OTA in the fluorescence detector was carried out at the wavelength of excitation of 335 nm and the wavelength of 465 nm.

### 2.6. Validation of the OTA Analysis

To validate the OTA analysis method, linearity, accuracy, precision, and sensitivity were determined. The limit of detection (LOD) and the limit of quantification (LOQ) were determined to identify the method’s sensitivity. The signal-to-noise ratios of 3:1 and 10:1 were considered for LOD and LOQ estimation. To obtain the analysis method’s accuracy, the recovery of OTA was measured. At first, the blank grape juice samples were spiked with OTA at 2.5, 5, and 10 µg/kg concentration levels. Then, OTA was extracted according to the method above. The recovery was calculated according to the following equation:

Recovery (%) = found OTA concentration/spiked OTA concentration × 100 (1)

The recovery test was repeated three times, and the relative standard deviation of three runs was calculated to show intra-day precision. For inter-day precision determination, the recovery test was performed on three consecutive days, and the relative standard deviation of nine repeats was calculated. To determine the linearity of the analysis method, the calibration curves were constructed using the peak area ratio of working standard solutions of OTA prepared at concentration levels of 0.1–25 µg/kg and analyzed by HPLC versus OTA concentration.

### 2.7. Measurement of Total Phenolic Acid Content

The total phenolic acid content (TPC) of the grape juice samples was determined according to the method suggested by Pankaj and Wan (2017) in a previous study [35]. Briefly, 0.5 mL of standard solution or sample was mixed with 1 mL of Folin–Ciocalteu reagent. After 6 min of incubation at room temperature, 2 mL of sodium carbonate solution (20%) was added. The mixture was placed for 60 min at 30 °C. Finally, the absorbance of the samples was recorded at 760 nm against the blank using a spectrophotometer (Shimadzu UV–Vis Mini 1240, Tokyo, Japan). The TPC was determined by a suitable calibration curve (6.25–100 μg/mL) and reported as μg of gallic acid equivalents/mL [35].

### 2.8. Measurement of Total Flavonoid Content

The method described by Pankaj et al. (2017) was used for determining the total flavonoid content (TFC) of samples [35]. At first, 0.25 mL of grape juice was mixed with 1.25 mL of deionized water and 75 μL of 5% sodium nitrate solution. After 6 min at room temperature, 150 μL of aluminum chloride solution (10%) was added to the mixture, and after 5 min, 0.5 mL of sodium hydroxide (1 M) was also added. Distilled water was used to adjust the total volume to 2.5 mL, and absorbance was observed at 415 nm by a spectrophotometer. The results were calculated and expressed as μg of catechin equivalents/mL using the calibration curve created from 12.5 to 100 μg/mL [35].

### 2.9. Antioxidant Capacity Measurement

The antioxidant potential of grape juice samples was measured via the Ferric Reducing Antioxidant Power Assay (FRAP) method described by Langley-Evans et al. (2000), with minor modifications [36]. Fresh FRAP reagent was composed of three solutions: acetate buffer (300 mmol/L) (pH 3.6), ferric chloride solution (20 mmol/L), and a solution of TPTZ (10 mmol/L) diluted in hydrochloric acid (40 mmol/L) at the ratio of 10:1:1 (*v*/*v*/*v*). Moreover, 50 µL of standard solution or grape juice to 700 µL reagent was added. In order to complete the reaction, the mixture was incubated for 5 min at 37 °C. Finally, the absorbance via a spectrophotometer was determined at 593 nm against a blank. The antioxidant power of the samples was detected from a plotted standard curve (62.5–1000 µM) and reported as µmoles of ferrous sulfate equivalents/L [36].

### 2.10. Experimental Design and Statistical Analysis of Data

Design of experiments and statistical analysis was performed using Design Expert 7.0.0 (Stat-Ease Inc., Minneapolis, Minnesota, USA). Response surface methodology (RSM), the face-centered central composite design (FCCD), was utilized for modeling and optimization of the influence of independent variables, including the three clarifiers, i.e., bentonite (X_1_), gelatin (X_2_), and diatomite earth (X_3_), at the levels of 0.25–0.75%, and clarifying time in levels of 1–3 h (X_4_) on dependent variables, i.e., the reduction amount (in %) of OTA, phenolic acid, flavonoids, and antioxidant capacity (FRAP assay). The mentioned levels were chosen by performing preliminary experiments, which indicated that clarifier concentrations (0.25–0.75%) and clarifying time (1–3 h) caused grape juice with desirable color quality. The experimental design included 30 experiments composed of 8-star points, 16 factorial points, and 6 center points (with four factors and three levels for each variable). The mentioned points and findings are shown in Table 1. The experimental data were fitted to the second-order polynomial equation:

Y = β_0_ + β_1_X_1_ + β_2_X_2_ +β_3_X_3_ + β_4_X_4_+ β_11_X_1_^2^ + β_22_X_2_^2^ + β_33_X_3_^2^ + β_44_X_4_^2^ + β_12_X_1_X_2_ + β_13_X_1_X_3_ + β_14_X_1_X_4_ + β_23_X_2_X_3_ + β_24_X_2_X_4_. (2)

where Y is the response; β_0_ is a constant coefficient; independent variables are (X_1_: concentration of bentonite, X_2_: concentration of gelatin, X_3_: concentration of diatomite, and X_4_: time of clarification); the coefficients of the equation are (β_1_–β_4_: the linear terms; β_11_–β_44_: the quadratic terms; and β_12_–β_24_ are the interaction terms). Analysis of Variance (ANOVA) was performed at the probability levels *p* < 0.05 and *p* < 0.01 to obtain the coefficients of the final equation for better accuracy.

### 2.11. Optimization

OTA’s reduction amount (in %) was kept at the maximum level for optimization. In contrast, the reduction amount (in %) of other responses, such as phenolic acid, flavonoids, and antioxidant capacity (FRAP assay) reduction content, was kept at the minimum value, and the independent variables (X_1_, X_2_, X_3_, X_4_) were placed within the range (between lower and higher level). The higher desirability value (0–1) was chosen among the optimum conditions suggested by the Design Expert software.

## 3. Results and Discussion

### 3.1. Method Validation

The results of experiments on the accuracy (recovery), precision (intra-day and inter-day), LOD, LOQ, and linearity of the analysis method for OTA measurement are presented in Table 2. The equation of the calibration curve was Y = 95,167X + 9867, with determination coefficients (R^2^) of 0.9995. Recovery values ranged from 96.09 to 101.23%, and the RSD of inter- and intra-day precision was lower than 20%. All of these results were in accordance with legal requirements (accuracy range: 70–110% and RSD < 20%) of EU regulations [37]. The LOQ value is well below the maximum allowable limit of OTA in juice (2 μg/kg) [22]. These findings showed that the validated method could be applied well for grape juice’s OTA determination.

### 3.2. OTA Content, Antioxidant Content, and Capacity of Unclarified Grape Juice

The average concentration of OTA, TPC, and TFC and the antioxidant capacity of grape juice before clarification was 5 µg/kg, 295.91 µg gallic acid/mL, 198.43 μg catechin/mL, and 1362.14 µmoles ferrous sulphate/L, respectively. The results of phenolic acid, flavonoid, antioxidant activity, and OTA reduction percentage during grape juice clarification are shown in Table 1.

### 3.3. The Effect of Grape Juice Clarification on the Reduction of OTA

The findings obtained by Design Expert software indicated that a linear (first-order) model could significantly predict the reduction percentage as a function of clarifier concentration and clarification time. This model was well fitted (*p* < 0.01, F-value = 16.96) and also showed a non-significant lack of fit (Table 3). The equation for OTA reduction percentage included:

The reduction of OTA (%) = 40.05 + 3.13X_1_ + 5.98X_2_ (3)

The highest percentage of OTA removal (50.56%) was related to the grape juice sample clarified for 3 h by 0.75% bentonite, 0.75% gelatin, and 0.75% diatomite. Moreover, the lowest removal of OTA (27.65%) was achieved in grape juice clarified for 1 h with 0.75% bentonite, 0.25% gelatin, and 0.75% diatomite (Table 1).

**Figure 1:**
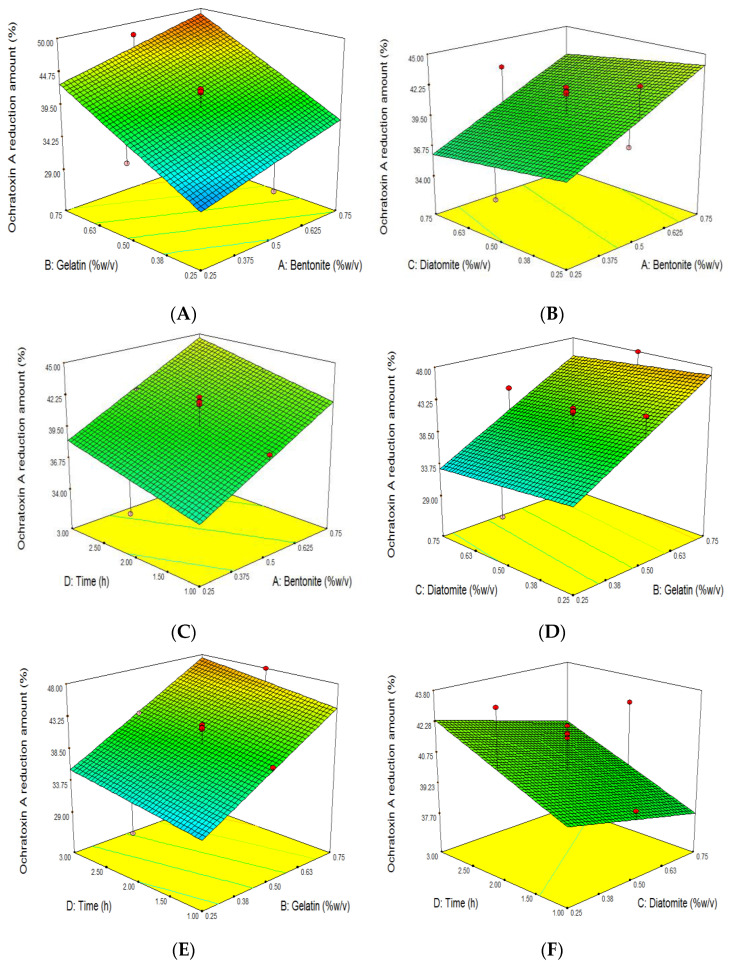
Response surface plot of the effects of bentonite, gelatin, and diatomite concentration and clarifying time on ochratoxin A reduction amount (%) of grape juice. Influence of (**A**): gelatin and bentonite, (**B**): diatomite and bentonite, (**C**): time and bentonite, (**D**): diatomite and gelatin, (**E**): time and gelatin, (**F**): time and diatomite, on ochratoxin A reduction amount (%).

With an increment in bentonite (X_1_) and gelatin (X_2_) levels from 0.25% to 0.75%, the loss of OTA was significantly increased (Figure 1A–E). Our findings are similar to those of previous studies that showed that increasing the concentration of bentonite in different types of wine led to a decrease in OTA [23,38,39]. However, Sun et al. (2017) observed that there were no significant differences among the OTA removal of wine samples clarified by various concentrations of bentonite (0.12, 0.16, and 0.20 mg/mL), and they could result in a 10% loss of OTA [29].

The formation of a hydrogen bond between OTA and bentonite could cause the removal of this mycotoxin [40]. In addition, bentonite has a layered structure [41], and protein-bound OTA is trapped within bentonite layers and separated from the grape juice matrix [12].

Among various clarifiers applied in this study, gelatin had the most significant influence on OTA reduction (Table 4). Previous studies reported the impact of gelatin on OTA reduction [38,40]. For example, Leong et al. (2006) found that an increment in gelatin concentration from 0.05% to 0.15% resulted in the OTA removal of wine being approximately increased from 13 to 21% [38], which follows our results. However, Castellari et al. (2001) found that OTA absorption was decreased with high concentrations of gelatin [40]. The OTA’s negative charge (carboxyl group of the phenylalanine moiety) interacts with positively charged gelatin (amino group) [39,40].

Among the three clarifiers, the minimum effect on OTA reduction was related to diatomite. As shown in Table 4, the effect of this independent variable on OTA is not significant (*p* > 0.05). However, diatomite has been applied in the juice production industry [42], and there is limited information about its effect on OTA. Lulamba et al. (2019) indicated that diatomite (200 mL/7gr) in beer and distilled water led to an OTA decrease to 38.4 and 17.9%, respectively. Diatomite has a layered structure, and OTA bonded with protein could be trapped in these layers [43]. Moreover, it is presumed that hydrogen exists in diatomite with the formula SiO_2_·nH_2_O, which plays a role in forming hydrogen bonds.

Based on Table 4, the influence of clarification time on OTA reduction was insignificant (*p* > 0.05), although, with a longer time, the removal of OTA was greater. Our results are similar to those of Sun et al.’s (2017) study. These authors found no significant difference in the clarification of different wine samples clarified by bentonite and gelatin [29].

### 3.4. The Effect of Grape Juice Clarification on the Reduction of TPC

The behavior of TPC during the grape juice clarification process has high importance because the phenolic compounds, such as resveratrol, have antioxidant, cardioprotective, antidiabetic, anticancer, and antiaging properties, and their removal causes nutritional loss in grape juice [44,45]. The experimental results demonstrated that the decrease in TPC followed the quadratic polynomial model (Table 3):

The reduction of TPC = 27.07 + 2.66X_1_ + 2.56X_2_ + 2.61X_3_ + 3.61X_4_ + 2.25X_1_^2^ + 1.87X_2_^2^ + 2.35X_3_^2^ (4)

The linear effects of all factors (*p* < 0.01) and the quadratic effects of bentonite, gelatin, and diatomite (*p* < 0.05) significantly influenced the decline in TPC, whereas the remaining terms were not significant (*p* > 0.05) (Table 4). The highest decline in TPC (44.71%) was obtained by using the following conditions: X_1_: 0.75%, X_2_: 0.75%, X_3_: 0.75%, and X_4_: 3 h. Meanwhile, the lowest reduction (20.8%) was achieved by utilizing the following parameters: X_1_: 0.25%, X_2_: 0.25%, X_3_: 0.25%, and X_4_: 1 h (Table 1).

There are various conflicting reports about the effect of bentonite on wine TPC. For example, some researchers suggested that enhancing bentonite increases TPC removal, which is in line with our studies [32,46]. Meanwhile, several authors presented contrasting results and mentioned that adding bentonite reduces TPC loss [47,48]. The platelets of bentonite carry a negative charge and can electrostatically bond to positively charged proteins that contain TPC and tannin [48,49,50]. In addition, cations located in bentonites such as Al^3+^, Ca^2+^, Mg^2+^, Mn^2+^, Zn^2+^, Cu^2+^, Fe^2+^, Na^+^, K^+^, and H^+^ interact directly with negatively charged phenolic acids in juice or wine [31].

As presented in Table 4, gelatin had a weak role in TPC removal because it has the lowest regression coefficients. The reduction of phenolic acid by gelatin was reported in other studies [46,47,51]. Because of the low pH of fruit juice, gelatin had a positive charge and could absorb negatively charged phenolic acid [32,51].

The reduction of TPC by diatomite has been documented [30,52]. For example, Capanoglu et al. (2013) found that the utilization of diatomite for grape juice filtration caused a reduction in TPC from 276 to 259 mg/g (approximately 6.16% loss) [30]. In another study, Fang et al. (2007) indicated that the application of diatomite as a filtering agent for bayberry juice led to a decrease of 2–5% in TPC [52]. It is thought that the hydrogen bond formation between the active silanol groups of diatomite and the hydroxyl groups of TPC could result in phenolic acid removal [31,53].

**Figure 2:**
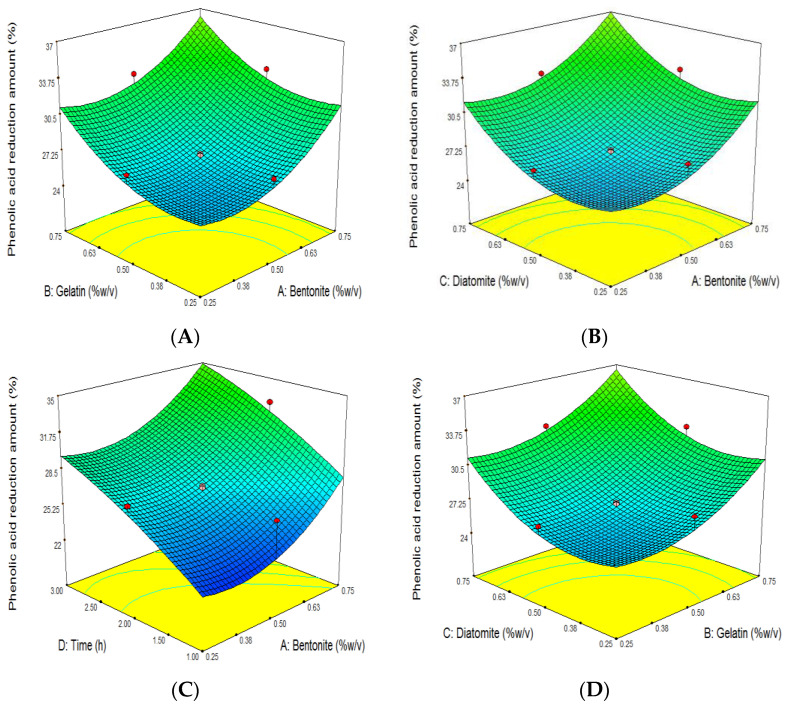

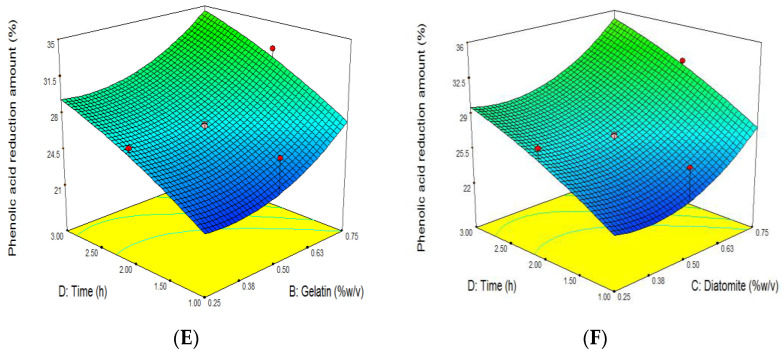
Response surface plot of the effects of bentonite, gelatin, and diatomite concentration and clarifying time on grape juice’s phenolic acid reduction amount (%). Influence of (**A**): gelatin and bentonite, (**B**): diatomite and bentonite, (**C**): time and bentonite, (**D**): diatomite and gelatin, (**E**): time and gelatin, (**F**): time and diatomite, on grape juice’s phenolic acid reduction amount (%).

When the clarifiers were in contact with grape juice for 3 h instead of 1 h, more TPC was removed (Figure 2C,E,F). Table 4 shows that the clarification time (X_4_) has the greatest influence on the decline in TPC from grape juice due to its higher coefficient compared to other factors.

### 3.5. The Effect of Grape Juice Clarification on the Reduction of TFC

The reduction of TFC ranged from 7.21% in the case of the experiments performed at the lowest level of clarifier amount and clarification time (Run 1 in Table 1) to 25.81% at the highest levels of clarifier amount and clarification time (Run 28 in Table 1). The ANOVA analysis indicated that the quadratic (second-order) model with a high coefficient of determination (R2 = 0.9282) fitted significantly (*p* < 0.01) to the reduction response of TFC (Table 3). In this model, the linear effects of bentonite, gelatin, diatomite, time (*p* < 0.01), and the quadratic effect of gelatin (*p* < 0.05) on the reduction of TFC are significant, whereas the residue of terms is not significant (*p* > 0.05) (Table 4). The multiple regression equation for the reduction of TFC is as follows:

The reduction of TFC (%) = 13.06 + 1.77X_1_ + 2.07X_2_ + 3.56X_3_ + 2.56X_4_ + 2.46X_2_^2^ (5)

**Figure 3:**
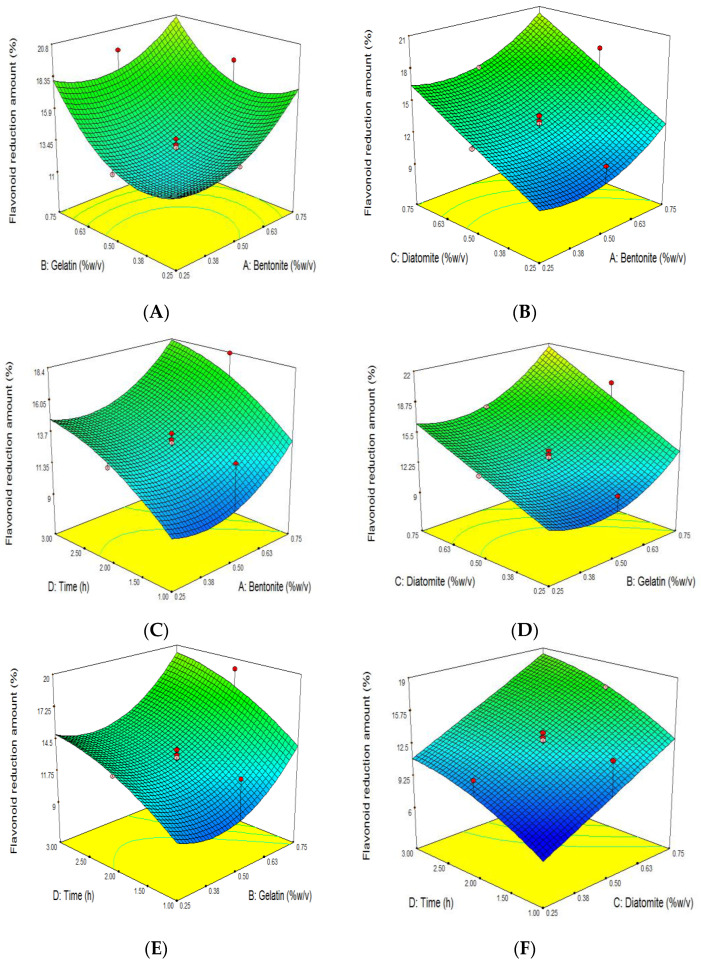
Response surface plot of the effects of bentonite, gelatin, and diatomite concentration and clarifying time on flavonoid reduction amount (%) of grape juice. Influence of (**A**): gelatin and bentonite, (**B**): diatomite and bentonite, (**C**): time and bentonite, (**D**): diatomite and gelatin, (**E**): time and gelatin, (**F**): time and diatomite, on grape juice’s flavonoid reduction amount (%).

Among the significant effects, bentonite has the least influence on the reduction of TFC compared to other effects (Table 4). The reduction of TFC was stable when adding bentonite (X_1_) and gelatin (X_2_) from 0.25 to 0.5%. However, increasing the bentonite and gelatin concentration to 0.75% improved TFC reduction (Figure 3A–E). Fang et al. (2007) expressed that the refining and filtration of wine with bentonite (0.2 g/L), gelatin (0.2 g/L), and diatomite (2 g/L) resulted in a decrease in TFC from 341.1 (mg/L) to 289.7 (mg/L) (approximately 15.07% reduction), which is within the range of our data [52]. However, the reduction of TFC (approximately 5% reduction) in mulberry wine clarified by bentonite was lower than in our findings [4]. It was proposed that hydrogen bonds between the benzene rings of anthocyanin and bentonite caused the complex formation and the removal of flavonoids, including anthocyanin [48].

The interaction effects of gelatin on the reduction of TFC with bentonite, diatomite, and time were investigated and are shown in Figure 3A,D,E. Among the second-order coefficients, gelatin has the highest value. Therefore, it showed the most substantial impact on the reduction of TFC (Table 4). Ren et al. (2020) found that the clarifying of berry wine with gelatin resulted in a reduction in TFC (approximately 16.5%) [54]. Anthocyanins and gelatin are positively charged; therefore, they could not absorb each other [50]. However, tannins containing a negative charge could play the role of mediator between both mentioned components and settle them.

With increasing diatomite (X_3_) from 0.25 up to 0.75%, the reduction of TFC was linearly increased (Figure 3B,D,F). In a study conducted by Capanoglu et al. (2013), the filtration of grape juice concentrates with diatomaceous earth resulted in the reduction of TFC from 122 to 15 mg/mL (approximately 87.7% reduction) [30]. In another study, the authors showed that diatomaceous earth caused the reduction of total anthocyanins of açai juice by 20.4% [33]. The positively charged anthocyanins in grape juice could flocculate with diatomite’s negatively charged hydroxyl group [50].

There is a direct relation between clarification time and the reduction of TFC (Figure 3C,E,F). Flavonoids are a type of active food constituent found in nature and are of particular interest because of their potential antioxidant activity and possible beneficial effects on human health [55,56]. As a result, their decrease during the clarification of grape juice must be minimized.

### 3.6. The Effect of Grape Juice Clarification on the Reduction of Antioxidant Capacity

Due to the antioxidative properties of grapes and their ability to reduce or prevent oxidative stress [57], the assessment of clarifying grape juice antioxidant capacity has high importance. In this study, the antioxidant capacity reduction (assessed by the FRAP method) of grape juice samples ranged between 16.43% (Run 1) and 19.89% (Run 17) (Table 1). For generating a relationship between the antioxidant capacity reduction and clarifier concentration, and clarification time (Table 3), a linear polynomial equation is given as follows:

Reduction of antioxidant capacity (%) = 18.13 + 0.35X_1_ + 0.34X_2_ + 0.51X_3_ + 0.43X_4_ (6)

**Figure 4:**
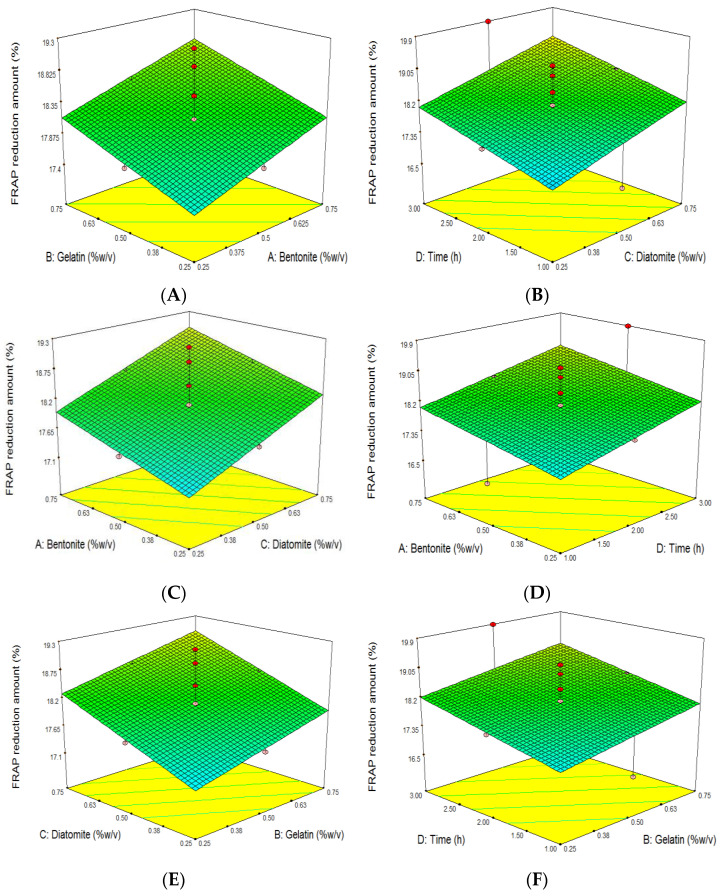
Response surface plot of the effects of bentonite, gelatin, and diatomite concentration and clarifying time on grape juice’s antioxidant capacity reduction amount (%). Influence of (**A**): gelatin and bentonite, (**B**): diatomite and bentonite, (**C**): time and bentonite, (**D**): diatomite and gelatin, (**E**): time and gelatin, (**F**): time and diatomite, on grape juice’s antioxidant capacity reduction amount (%).

The increase in clarifier agent concentration from 0.25 to 0.75% was positively associated with antioxidant activity reduction (Figure 4A–F). Our data agree with those of other researchers who used bentonite in different concentrations for the clarification of red wine and showed that a diminution in antioxidant content is linked to an increase in bentonite [46,58]. In contrast to our findings, Ghanem et al. (2017) observed that an increment in bentonite value from 450 up to 800 mg/L resulted in an enhancement in the antioxidant capacity of red wine (2.90–2.92 mg/mL) [32]. It seems that bentonite absorbs some phenols involved in antioxidant activity, such as catechin and caffeic acid [31].

The lowest effect on reducing antioxidant activity among the three clarifiers was shown by gelatin (Table 4). Some authors indicated that gelatin causes red wine’s antioxidant capacity, similar to our results [46,58]. In contrast to these findings, Ghanem et al. (2017) demonstrated that wine samples clarified with a high gelatin concentration had greater antioxidant capacity than those with a low level of this clarifier [32]. Dıblan et al. (2021) found that the binding of phenolic acid as an antioxidant compound to protein clarifiers such as gelatin could decrease the antioxidant capacity of grape juice [31].

Diatomite, with a high coefficient, has the most significant effect on reducing antioxidant capacity (Table 4). In order to manufacture grape juice concentrate, Capanoglu et al. (2013) employed diatomite for filtration and reported that it diminished antioxidant activity from 439 to 378 µmol/g (approximately 13.9%) [30]. On the other hand, according to Farahmand et al. (2017), diatomite filtration reduces the antioxidant content of pomegranate juice from 8 to 7.1 µg/mL (approximately 11.25%) [59]. It is explained that some antioxidant components in grape juice, such as anthocyanin, have a positive charge and can flocculate with negatively charged diatomite [50,60].

As seen in Figure 4B,D,F, the elimination of antioxidant activity can be improved by expanding the clarifier’s contact time (X_4_) with grape juice from 1 to 3 h. To our knowledge, no data exist on clarification time’s influence on antioxidant activity.

### 3.7. Process Optimization

**Figure 5:**
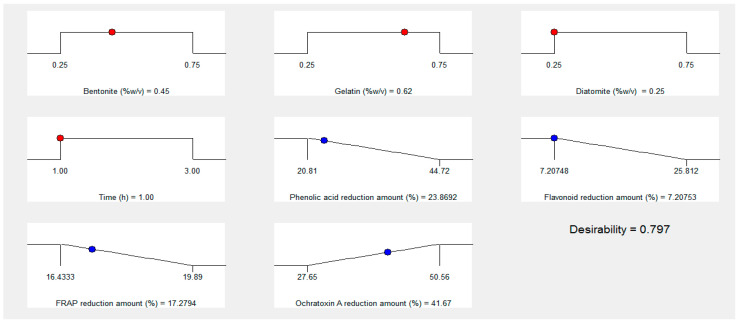
The optimum value of different variables for OTA removal of grape juice.

In order to produce safe grape juice, multi-objective optimization was employed to achieve maximum OTA diminution and a minimal decrement in compounds and antioxidant capacity. After analyzing the data of thirty experiments and obtaining valid prediction models for each response, the software selected a solution. As shown in Figure 5, the optimal experimental conditions predicted by the face-centered central composite design were: bentonite of 0.45% *w*/*v*, gelatin of 0.62% *w*/*v*, diatomite of 0.25 *w*/*v*, and clarification time of 1 h. The reductions in OTA, TPC, TFC, and antioxidant activity were calculated as 41.67%, 23.86%, 7.20%, and 17.27%, respectively.

## 4. Conclusions

Clarification is the most crucial step in the process of grape juice manufacture. This study employed prevalent clarifiers (bentonite, gelatin, and diatomite) to clarify grape juice. Moreover, the response surface methodology was used to find an optimal point. Bentonite and gelatin significantly affected the OTA level, whereas compounds and antioxidant capacity were significantly affected by all variables. In general, when enhancing the clarifier concentration (0.25–0.75%) and clarifying time (1–3 h), the removal of OTA, antioxidant compounds, and capacity increased. For the first time, in this report, the optimal values of grape juice clarification parameters with a desirability value of 0.797 to achieve the maximum reduction in OTA and the minimum reduction in compounds and antioxidant capacity were determined. The present study’s results encourage more research to produce healthy grape juice.

## Figures and Tables

**Table 1:** Experimental design and results of OTA, phenolic acid, flavonoid, and antioxidant activity reduction percentage during grape juice clarification.

Run	Independent Variables					Dependent Variables						
	X_1_: Bentonite (% *w*/*v*)	X_2_: Gelatin (% *w*/*v*)	X_3_: Diatomite (% *w*/*v*)	X_4_: Time (h)	OTA (µg/kg)	Loss of OTA (%)	TPC (µg/mL)	Loss of TPC (%)	TFC (µg/mL)	Loss of TFC (%)	FRAP (µmol/L)	Loss of FRAP (%)
1	0.25 (−1)	0.25 (−1)	0.25 (−1)	1 (−1)	3.47	30.56	234.33	20.81	184.13	7.21	1138.34	16.43
2	0.25 (−1)	0.25 (−1)	0.75 (+1)	1 (−1)	3.37	32.65	218.68	26.10	173.6	12.51	1122.40	17.6
3	0.25 (−1)	0.75 (+1)	0.25 (−1)	1 (−1)	3.07	38.55	217.94	26.35	177.91	10.34	1126.76	17.28
4	0.25 (−1)	0.75 (+1)	0.75 (+1)	1 (−1)	3.00	39.95	202.91	31.43	164.16	17.27	1113.28	18.27
5	0.25 (−1)	0.5 (0)	0.5 (0)	2 (0)	3.30	34.00	214.68	27.45	173.21	12.71	1120.77	17.72
6	0.25 (−1)	0.25 (−1)	0.25 (−1)	3 (+1)	3.50	30.06	210.66	28.81	174.69	11.96	1128.67	17.14
7	0.25 (−1)	0.25 (−1)	0.75 (+1)	3 (+1)	3.40	32.1	194.86	34.15	164.16	17.27	1115.05	18.14
8	0.25 (−1)	0.75 (+1)	0.25 (−1)	3 (+1)	2.74	45.15	194.56	34.25	168.26	15.20	1119.54	17.81
9	0.25 (−1)	0.75 (+1)	0.75 (+1)	3 (+1)	2.71	45.85	179.00	39.51	147.31	25.76	1105.92	18.81
10	0.5 (0)	0.5 (0)	0.5 (0)	1 (−1)	3.05	39.00	218.29	26.23	172.55	13.04	1137.11	16.52
11	0.5 (0)	0.5 (0)	0.5 (0)	2 (0)	3.10	38.00	221.58	25.12	176.08	11.26	1116.41	18.04
12	0.5 (0)	0.5 (0)	0.5 (0)	2 (0)	3.09	38.24	216.81	26.73	171.41	13.62	1116.27	18.05
13	0.5 (0)	0.5 (0)	0.5 (0)	2 (0)	2.92	41.7	222.08	24.95	176.55	11.03	1115.59	18.1
14	0.5 (0)	0.5 (0)	0.5 (0)	2 (0)	2.90	42.10	216.34	26.89	172.8	12.92	1104.83	18.89
15	0.5 (0)	0.5 (0)	0.5 (0)	2 (0)	2.93	41.50	215.96	27.02	172.37	13.13	1101.29	19.15
16	0.5 (0)	0.5 (0)	0.5 (0)	2 (0)	2.91	41.75	216.58	26.81	174.04	12.29	1110.83	18.45
17	0.5 (0)	0.5 (0)	0.5 (0)	3 (+1)	2.94	41.15	212.05	28.34	173.47	12.58	1091.21	19.89
18	0.75 (+1)	0.25 (−1)	0.75 (+1)	1 (−1)	3.62	27.65	202.34	31.62	164.16	17.27	1113.14	18.28
19	0.5 (0)	0.25 (−1)	0.5 (0)	2 (0)	3.52	29.60	215.6	27.14	172.08	13.28	1120.77	17.72
20	0.5 (0)	0.5 (0)	0.25 (−1)	2 (0)	2.81	43.80	212.91	28.05	176.34	11.13	1123.22	17.54
21	0.5 (0)	0.5 (0)	0.75 (+1)	2 (0)	2.88	42.45	199.98	32.42	165.82	16.43	1109.60	18.54
22	0.5 (0)	0.75 (+1)	0.5 (0)	2 (0)	2.60	47.97	200.15	32.36	160.48	19.13	1111.64	18.39
23	0.75 (+1)	0.25 (−1)	0.25 (−1)	1 (−1)	2.83	43.35	216.43	26.86	180.04	9.27	1126.76	17.28
24	0.75 (+1)	0.25 (−1)	0.25 (−1)	3 (+1)	2.79	44.23	194.83	34.16	166.19	16.25	1119.54	17.81
25	0.75 (+1)	0.75 (+1)	0.75 (+1)	1 (−1)	2.54	49.23	187.22	36.73	156.86	20.95	1104.15	18.94
26	0.75 (+1)	0.75 (+1)	0.25 (−1)	1 (−1)	2.56	48.75	203.35	31.28	173.61	12.51	1117.50	17.96
27	0.75 (+1)	0.75 (+1)	0.25 (−1)	3 (+1)	2.53	49.32	180.95	38.85	161.72	18.50	1110.42	18.48
28	0.75 (+1)	0.75 (+1)	0.75 (+1)	3 (+1)	2.47	50.56	163.58	44.72	147.21	25.81	1096.80	19.48
29	0.75 (+1)	0.25 (−1)	0.75 (+1)	3 (+1)	3.12	37.54	178.49	39.68	152.55	23.12	1105.65	18.83
30	0.75 (+1)	0.5 (0)	0.5 (0)	2 (0)	3.27	34.67	198.82	32.81	162.03	18.34	1138.34	18.39

OTA: ochratoxin A, TPC: total phenolic content, TFC: total flavonoid content, FRAP: Ferric Reducing Antioxidant Power.

**Table 2:** Data regarding method validation.

Spiked Concentration (µg/kg)	Recovery				Linearity (µg mL^−1^)	LOD (µg kg^−1^)	LOQ (µg kg^−1^)
	Intra-Day	RSD	Inter-Day	RDS			
2.5	98.25	12.56	99.78	14.87			
5	101.23	11.87	100.85	15.87	0.05–25	0.07	0.23
10	97.54	9.87	96.09	10.76			

**Table 3:** ANOVA for responses by response surface method.

Response	Source	Sum of Square	DF	Mean Square	F Value	Probe > F	Model
OTA reduction amount (%)	Model	872.19	4	218.05	16.96	<0.0001	Linear
	Lack of fit	303.42	20	15.17	4.24	0.0580	
	Pure error	17.91	5	3.58			
	Total	49,307.99	30	1643.60			
	R^2^						0.7308
TPC reduction amount (%)	Model	207.72	4	51.93	26.57	<0.0001	Quadratic
	Lack of fit	24.80	10	2.48	2.75	0.1380	
	Pure error	4.51	5	0.90			
	Total	28,908.91	30	963.63			
	R^2^						0.9651
TFC reduction amount (%)	Model	78.65	4	19.66	6.73	0.0026	Quadratic
	Lack of fit	38.24	10	3.83	3.51	0.0892	
	Pure error	5.47	5	1.09			
	Total	7423.38	30	247.45			
	R^2^						0.9282
FRAP reduction amount (%)	Model	12.38	4	3.09	14.14	<0.001	Linear
	Lack of fit	4.34	20	0.22	0.96	0.5809	
	Pure error	1.13	5	0.23			
	Total	9880.13	30	329.34			
	R^2^						0.6935

**Table 4:** Regression coefficients of coded factors for the responses during the optimization of grape juice clarification.

Coefficients	Reduction of OTA (%)	*p*-Value	Reduction of TPC (%)	*p*-Value	Reduction of TFC (%)	*p*-Value	Reduction of FRAP (%)	*p*-Value
Intercept (X_0_)	+40.05	-	+27.07	-	+13.06	-	+18.13	-
X_1_	+3.14	0.0010	+2.66	<0.0001	+1.77	0.0005	+0.35	0.0042
X_2_	+5.98	<0.0001	+2.56	<0.0001	+2.07	0.0001	+0.34	0.0045
X_3_	−0.88	0.3092	+2.61	<0.0001	+3.56	<0.0001	+0.51	0.0001
X_4_	+1.46	0.0965	+3.61	<0.0001	+2.56	<0.0001	+0.43	0.0006
X_1_X_2_	-	-	−0.15	0.6726	−0.49	0.2735	-	-
X_1_X_3_	-	-	+0.039	0.9118	+0.16	0.7159	-	-
X_1_X_4_	-	-	−0.069	0.8453	+0.051	0.9073	-	-
X_2_X_3_	-	-	+0.047	0.8951	+0.48	0.2742	-	-
X_2_X_4_	-	-	+8.125 × 10^−3^	0.9818	+0.12	0.7888	-	-
X_3_X_4_	-	-	+0.088	0.8044	+0.086	0.8431	-	-
X_1_^2^	-	-	+2.25	0.0207	+1.78	0.1135	-	-
X_2_^2^	-	-	+1.87	0.0484	+2.46	0.0350	-	-
X_3_^2^	-	-	+2.35	0.0162	+0.040	0.9703	-	-
X_4_^2^	-	-	−0.60	0.5012	−0.93	0.3938	-	-

## Data Availability

The data obtained from the study are presented and discussed in the manuscript.
